# Therapeutic Efficacy of Vectored PGT121 Gene Delivery in HIV-1-Infected Humanized Mice

**DOI:** 10.1128/JVI.01925-17

**Published:** 2018-03-14

**Authors:** Alexander Badamchi-Zadeh, Lawrence J. Tartaglia, Peter Abbink, Christine A. Bricault, Po-Ting Liu, Michael Boyd, Marinela Kirilova, Noe B. Mercado, Ovini S. Nanayakkara, Vladimir D. Vrbanac, Andrew M. Tager, Rafael A. Larocca, Michael S. Seaman, Dan H. Barouch

**Affiliations:** a Center for Virology and Vaccine Research, Beth Israel Deaconess Medical Center, Harvard Medical School, Boston, Massachusetts, USA; b Ragon Institute of Massachusetts General Hospital, Massachusetts Institute of Technology and Harvard University, Cambridge, Massachusetts, USA; c Center for Immunologic and Inflammatory Diseases, Ragon Institute, Massachusetts General Hospital, Boston, Massachusetts, USA; Emory University

**Keywords:** HIV-1, PGT121, adeno-associated virus, adenoviruses, neutralizing antibodies

## Abstract

Broadly neutralizing antibodies (bNAbs) are being explored for HIV-1 prevention and cure strategies. However, administration of purified bNAbs poses challenges in resource-poor settings, where the HIV-1 disease burden is greatest. *In vivo* vector-based production of bNAbs represents an alternative strategy. We investigated adenovirus serotype 5 (Ad5) and adeno-associated virus serotype 1 (AAV1) vectors to deliver the HIV-1-specific bNAb PGT121 in wild-type and immunocompromised C57BL/6 mice as well as in HIV-1-infected bone marrow-liver-thymus (BLT) humanized mice. Ad5.PGT121 and AAV1.PGT121 produced functional antibody *in vivo*. Ad5.PGT121 produced PGT121 rapidly within 6 h, whereas AAV1.PGT121 produced detectable PGT121 in serum by 72 h. Serum PGT121 levels were rapidly reduced by the generation of anti-PGT121 antibodies in immunocompetent mice but were durably maintained in immunocompromised mice. In HIV-1-infected BLT humanized mice, Ad5.PGT121 resulted in a greater reduction of viral loads than did AAV1.PGT121. Ad5.PGT121 also led to more-sustained virologic control than purified PGT121 IgG. Ad5.PGT121 afforded more rapid, robust, and durable antiviral efficacy than AAV1.PGT121 and purified PGT121 IgG in HIV-1-infected humanized mice. Further evaluation of vector delivery of HIV-1 bNAbs is warranted, although approaches to prevent the generation of antiantibody responses may also be required.

**IMPORTANCE** Broadly neutralizing antibodies (bNAbs) are being explored for HIV-1 prevention and cure strategies, but delivery of purified antibodies may prove challenging. We investigated adenovirus serotype 5 (Ad5) and adeno-associated virus serotype 1 (AAV1) vectors to deliver the HIV-1-specific bNAb PGT121. Ad5.PGT121 afforded more rapid, robust, and durable antiviral efficacy than AAV1.PGT121 and purified PGT121 IgG in HIV-1-infected humanized mice.

## INTRODUCTION

The therapeutic efficacy of potent neutralizing human immunodeficiency virus type 1 (HIV-1)-specific broadly neutralizing antibodies (bNAbs) has been shown in both humanized mice (1) and simian-human immunodeficiency virus (SHIV)-infected rhesus monkeys (2) and is under investigation in HIV-1-infected humans (3–7). A single infusion of the glycan-dependent monoclonal antibody PGT121 also significantly reduced viral load in chronically SHIV-infected rhesus monkeys and converted low-viremia animals into “elite controllers” (2, 8).

Despite the therapeutic potential of potent HIV-1 bNAbs, there are challenges to the administration of purified antibodies in the developing world. These include (i) the necessity of repeated intravenous or subcutaneous dosing (9), (ii) high concentrations that may be required to achieve lasting clinical efficacy and subsequent high manufacturing costs (10), and (iii) temperature-controlled storage and distribution networks, which may not be available in the developing world (11). Given these challenges, alternative strategies that enable the production of bNAbs *in vivo* should be explored.

Here we investigate the use of recombinant adenoviruses (Ads) and adeno-associated viruses (AAVs) that encode the HIV-1-specific bNAb PGT121 to produce the therapeutic monoclonal antibody (MAb) *in vivo*. Ads encoding MAbs have previously shown therapeutic efficacy against infectious diseases, including Yersinia pestis (12), respiratory syncytial virus (13), and West Nile virus (14), as well as against chronic illnesses, including cancers (15), but Ads expressing HIV-1-specific bNAbs have not previously been evaluated. AAVs encoding HIV-1-specific bNAbs have been explored for long-term *in vivo* antibody expression and have been studied in mice (16), nonhuman primates (17), and humans (18). In this study, we show that both Ad5 and AAV1 produce functional PGT121 *in vivo*. Moreover, in HIV-1-infected humanized mice, Ad5.PGT121 produced PGT121 more quickly and afforded greater antiviral efficacy than did AAV1.PGT121.

## RESULTS

### Ad- and AAV-vectored gene delivery of PGT121.

**FIG 1:**
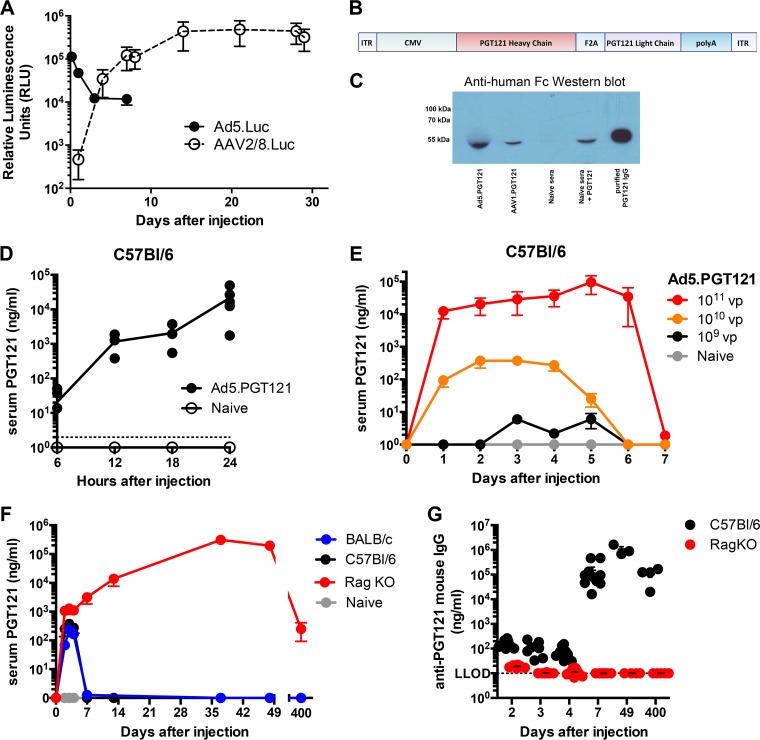
*In vivo* kinetics of Ad-vectored PGT121. (A) *In vivo* luciferase transgene expression in BALB/c mice following intramuscular injection of either Ad5.Luc (10^10^ vp) or AAV2/8.Luc (10^10^ vp) by IVIS imaging. (B) PGT121 expression cassette between AAV2 inverted terminal repeats (ITRs). Ad vectors do not contain ITRs. (C) Anti-human Fc receptor Western blot of sera from Ad5.PGT121- and AAV1.PGT121-injected mice, naive serum (negative control), naive serum spiked with purified PGT121 (positive control), and purified PGT121 alone (positive control). (D, E, F) Serum PGT121 concentrations measured by quantitative ELISA. (G) Serum anti-PGT121 antibody (mouse IgG) concentrations measured by quantitative ELISA. The dotted line represents the lower limit of detection (LLOD) for the assay. Each dot represents an individual mouse. *n* = 3 to 8 per group per experiment. Data are presented as means ± standard errors of the means (SEM).

We first sought to investigate the kinetics of *in vivo* PGT121 expression from Ad5 and AAV vectors. BALB/c mice were injected intramuscularly (i.m.) once with either 10^10^ viral particles (vp) of Ad5 expressing luciferase (Ad5.Luc) or 10^10^ genome copies (GC) of AAV2/8 expressing luciferase (AAV2/8.Luc), and luciferase expression was measured by IVIS imaging following viral injection. Ad5.Luc induced high levels of expression within 6 h, but expression levels then declined markedly by day 7 (Fig. 1A). In contrast, AAV2/8.Luc yielded low transgene expression (<10^3^ relative luminescence units [RLU]) on day 1, followed by increased expression by day 4 and a plateau of >10^5^ RLU from day 14 onwards (Fig. 1A).

Based on these different expression kinetics from Ad and AAV vectors, we constructed Ad5 and AAV1 vectors expressing the HIV-1 bNAb PGT121 (Fig. 1B). Expression of PGT121 from the Ad5 and AAV1 vectors *in vivo* was confirmed by a comparison of an anti-human Fc Western blot using serum samples of mice injected with Ad5.PGT121 or AAV1.PGT121 with a blot using naive serum spiked with purified PGT121 IgG (Fig. 1C).

We next monitored longitudinal *in vivo* PGT121 expression from Ad5.PGT121. C57BL/6 mice were injected i.m. once with 10^11^ vp of Ad5.PGT121, and serum samples were analyzed at 6, 12, 18, and 24 h after injection. The presence of PGT121 antibody in the serum was detectable as early as 6 h after Ad5.PGT121 injection (∼10 ng/ml), reaching a concentration in serum of ∼1 μg/ml at 12 h and >10 μg/ml by 24 h (Fig. 1D). PGT121 concentration was dose dependent (Fig. 1E). However, at all Ad5.PGT121 dosages, serum PGT121 levels declined to undetectable levels by day 7. This was also observed in BALB/c mice (Fig. 1F). In contrast, i.m. injection of Ad5.PGT121 (10^10^ vp) in Rag knockout (KO) mice yielded sustained levels of serum PGT121 for over 400 days (Fig. 1F).

Given the immunogenicity of human IgG in mice, we assessed the host anti-PGT121 antibody responses (19, 20). As expected, C57BL/6 mice raised potent anti-PGT121 responses by day 7, while Rag KO mice raised no detectable anti-PGT121 antibodies for over 400 days after Ad5.PGT121 injection (Fig. 1G), thus accounting for the sustained PGT121 expression in immunocompromised mice.

### Virus-vectored PGT121 has affinity and neutralization ability comparable to those of purified PGT121 IgG.

**FIG 2:**
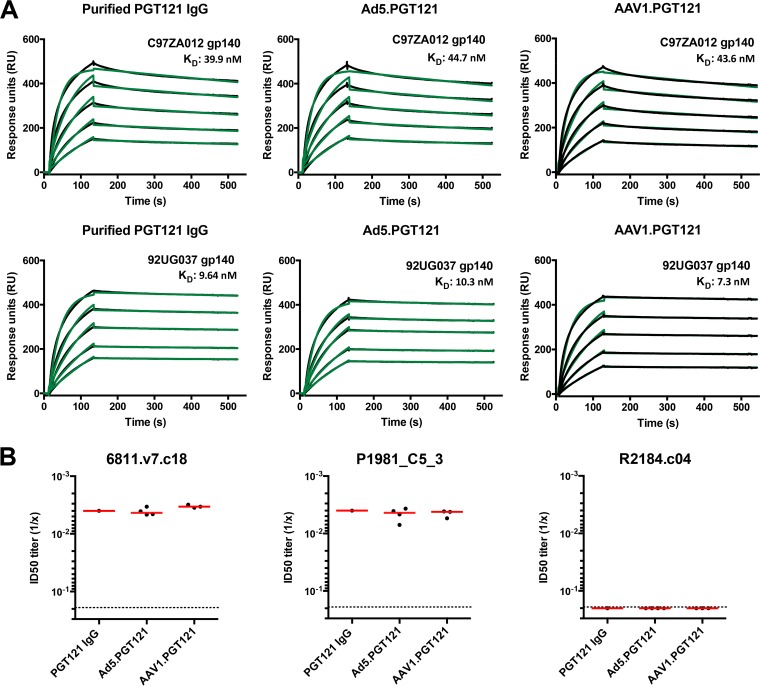
Functional characteristics of Ad- and AAV-vectored PGT121. (A) SPR binding profiles of gp140 (from C97ZA012 and 92UG037) to PGT121, from purified PGT121 IgG, or from sera of Ad5.PGT121- or AAV1.PGT121-injected mice. Protein A was irreversibly coupled to a CM5 chip, and IgGs were captured. Gp140 protein was allowed to flow over bound PGT121 IgG at concentration ranges from 62.5 to 1,000 nM. Raw sensograms are presented in black, and kinetic fits are in green. *K_D_*, equilibrium dissociation constant (in nanomolar units). (B) ID_50_ (50% inhibitory dilution) of serum PGT121 (Ad5 vectored, AAV1 vectored, and naive serum spiked with purified IgG) against Env-expressing pseudoviruses 6811.v7.c18 (clade CD), P1981_C5_3 (clade G), and R2184.c04 (clade CRF01_AE) (PGT121-resistant control). Each dot represents an individual mouse, and the red line indicates the mean. The dotted line denotes the limit of detection for the neutralization assay.

To assess whether the Ad5- and AAV1-produced PGT121 bound the HIV-1 envelope (Env) gp140 protein similarly to purified PGT121, we performed surface plasmon resonance (SPR). Ad5- and AAV1-expressed PGT121 bound C97ZA012 and 92UG037 gp140s with affinity comparable to that of purified PGT121 IgG (Fig. 2A). To measure functional capacity, we assessed the ability of Ad5- and AAV1-expressed PGT121 to neutralize selected HIV-1 gp160-expressing pseudoviruses. Vector-expressed PGT121 yielded mean 50% inhibitory dilution (ID_50_) titers similar to those of purified PGT121 IgG against two PGT121-sensitive pseudoviruses (6811.v7.c18 and P1981_C5_3) and one PGT121-resistant pseudovirus (R2184.c04) (Fig. 2B). These data suggest that Ad5- and AAV1-produced PGT121 was biochemically and functionally equivalent to purified PGT121 IgG.

### PGT121 expression in C57BL/6 and Rag KO mice.

**FIG 3:**
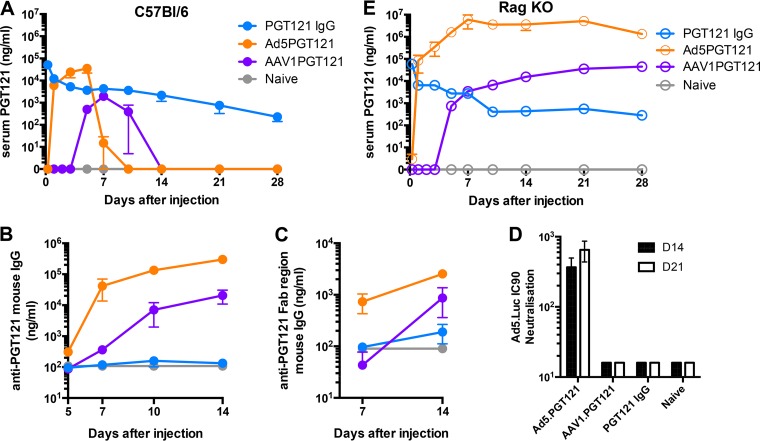
*In vivo* kinetics of vectored and nonvectored PGT121. (A and E) Serum PGT121 concentrations in C57BL/6 mice (A) and RagKO mice (E) measured by quantitative ELISA. (B and C) Serum anti-PGT121 antibody (mouse IgG) (B) and serum anti-PGT121 Fab region (mouse IgG) (C) concentrations measured by quantitative ELISA. (D) Serum 90% neutralization capacity (IC_90_) of mouse serum against Ad5 at days 14 and 21 after vector injections. *n* = 4 to 5 per group per experiment. Data are presented as means ± SEM.

We next sought to investigate the pharmacokinetics of Ad5-vectored, AAV1-vectored, and purified PGT121 IgG in immunocompetent and immunosuppressed mice. C57BL/6 mice were injected i.m. with Ad5.PGT121 (10^11^ vp) or AAV1.PGT121 (10^11^ vp) or received by intravenous (i.v.) infusion 20 μg purified PGT121 IgG, and serum PGT121 concentrations were measured by enzyme-linked immunosorbent assay (ELISA) (Fig. 3A and E). In immunocompetent C57BL/6 mice, Ad5.PGT121 yielded detectable levels of PGT121 by day 1 and peaked at concentrations in serum of 35 μg/ml PGT121 on day 5 before precipitously declining by days 7 to 10. As previously observed (Fig. 1A and G), AAV1.PGT121 exhibited a delay in detectable levels of serum PGT121 until day 5, peaking on day 7, and then declining by days 10 to 14. Infusion of purified PGT121 IgG resulted in detectable but declining serum PGT121 levels for >28 days.

All immunocompetent mice that received Ad5.PGT121 induced potent anti-PGT121 antibodies by day 7. AAV1.PGT121 elicited slightly delayed anti-PGT121 antibodies by days 7 to 10. Purified PGT121 did not elicit detectable levels of anti-PGT121 antibodies for up to 14 days (Fig. 3B). Thus, the reduction of serum PGT121 levels in AAV1.PGT121- and Ad5.PGT121-treated mice coincided with the development of anti-PGT121 antibodies. To investigate whether the anti-PGT121 antibodies raised were targeted against a specific region of PGT121, we ran additional quantitative ELISAs against the PGT121 Fab. The same trend was observed, although anti-Fab PGT121 antibodies were of lower magnitude than total anti-PGT121 antibodies (Fig. 3C). Ad5.PGT121 also elicited anti-Ad5 vector-specific neutralizing antibodies within 14 days (Fig. 3D).

In contrast, in Rag KO mice, Ad5.PGT121 produced detectable levels of PGT121 by day 1, and serum PGT121 levels were then durably sustained at the remarkably high levels of approximately 6 mg/ml for >28 days. AAV1.PGT121 exhibited a delay in detectable PGT121 levels until day 5 and was sustained at levels of approximately 44 μg/ml. Intravenous injection of purified PGT121 IgG resulted in detectable serum PGT121 with declining levels over 28 days, similar to what was observed with immunocompetent mice (Fig. 3E). No anti-PGT121 or anti-Ad5 antibodies were observed in Rag KO mice (data not shown). Anti-PGT121 antibody titers coincided with reduced PGT121 IgG levels, but we cannot rule out a potential suppressive role of cellular responses as well (21).

### Ad5.PGT121 rapidly reduces and maintains a lower viral load in HIV-1-infected humanized mice.

**FIG 4:**
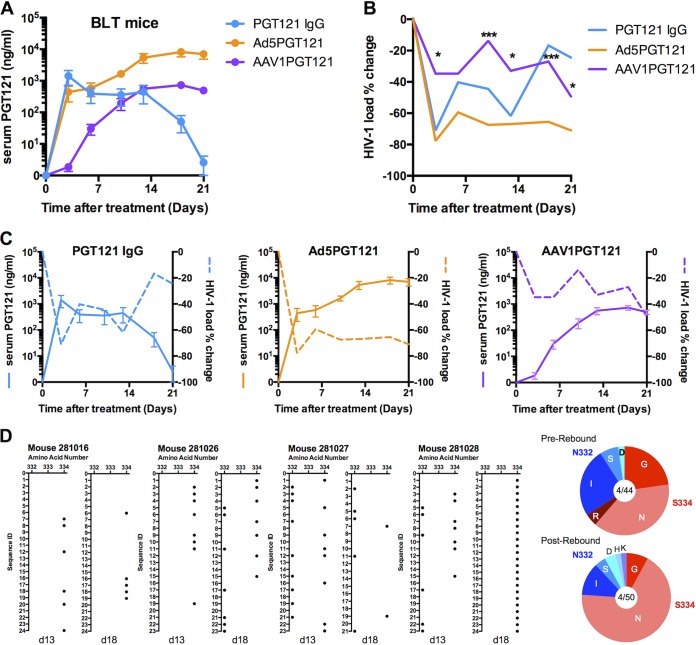
Vectored PGT121 reduces viral loads in HIV-1-infected humanized mice. (A and B) Serum PGT121 concentrations (A) and log plasma HIV-1 viral RNA (copies/ml) (B) in BLT mice following vectored PGT121 or PGT121 IgG treatment. HIV-1 viral load percentage changes from peak load 1 day prior to treatment (D-1) to day 21 after vectored PGT121 or PGT121 IgG treatment. (C) Serum PGT121 concentration plotted against HIV-1 viral load percentage decreases following therapeutic intervention. (D) Single-genome amplification (SGA) sequence analysis of HIV-1 gp120 pre- and postviral rebound following therapy with intravenous PGT121 IgG. The dot graph shows the distribution and frequency of amino acid mutations (black dot) before (day 13) and after (day 18) viral rebound. Pie charts show the frequency of amino acid changes in the 4 mice over a number of gp120 sequences for pre- and postrebound samples. Mutations are relative to the wild-type sequence of HIV-1 JR-CSF. *n* = 4 per group. Serum PGT121 concentrations are presented as means ± SEM. Data represent mean viral loads. *, *P* < 0.05; ***, *P* < 0.001 (Mann-Whitney *U* test).

To assess the therapeutic potential of Ad5.PGT121 and AAV1.PGT121 *in vivo*, 12 BLT (bone marrow/liver/thymus) humanized mice were infected with HIV-1 strain JR-CSF. HIV-1 infection was confirmed in all mice after infection, with stable setpoint viral loads prior to PGT121 administration (data not shown). Ad5.PGT121 (10^11^ vp i.m.), AAV1.PGT121 (10^11^ vp i.m.), and PGT121 IgG (20 μg i.v.) were administered on day 17 (*n* = 4/group). Serum PGT121 concentrations following the treatments in BLT mice showed kinetics similar to those of Rag KO mice (Fig. 4A) (22). Serum HIV-1 viral loads were determined by reverse transcription-PCR (RT-PCR). Ad5.PGT121 and purified PGT121 IgG resulted in substantial reductions of viral loads, whereas AAV1.PGT121 had a more modest effect, likely due to lower and delayed PGT121 levels. Ad5.PGT121 treatment reduced the mean HIV-1 viral load by 78% compared with baseline, from 1.27 ×10^6^ to 2.78 ×10^5^ RNA copies/ml (Fig. 4B). In contrast, AAV1.PGT121 reduced viral loads to a lesser extent than did Ad5.PGT121 (*, *P* < 0.05; ***, *P* < 0.001) (Fig. 4B).

Serum PGT121 levels following Ad5.PGT121, AAV1.PGT121, and PGT121 IgG administration correlated with the reductions in viral loads, suggesting that serum PGT121 levels of >100 ng/ml are required to suppress HIV-1 replication in this model (Fig. 4C). Soluble PGT121 IgG led to an early but transient viral load decline, but this effect was lost by days 14 to 21, presumably due to declining serum PGT121 levels. Thus, Ad5.PGT121 may offer advantages over both AAV1.PGT121 and PGT121 IgG in this model, as a result of its rapid, robust, and sustained PGT121 expression.

In the PGT121 IgG-treated mice, the decline of serum PGT121 levels after day 14 correlated with viral rebound (Fig. 4C). To investigate whether the viral rebound was due to the development of HIV-1 JR-CSF escape mutants, we performed single-genome amplification (SGA) and sequencing on day 13 (prerebound) and day 18 (postrebound). Following rebound, there was no consistent increase in mutations at residues 332 and 334, which correspond to the potential *N*-linked glycosylation site (PNGS) in the V3 loop (23). Some S334N mutations were observed, and in one mouse (281028), all 24 rebound sequences revealed the S334N mutation (Fig. 4D). Prior research has revealed that mutations at residues 332 and 334 in SHIV-SF162P3 only partially reduce the neutralization activity of PGT121 IgG (24), presumably as a result of the ability of PGT121 to make multiple glycan contacts on HIV-1 Env (23).

## DISCUSSION

In this study, we demonstrate that the HIV-1 bNAb PGT121 expressed by Ad5 was functional and capable of reducing viral loads *in vivo* in HIV-1-infected humanized mice. Ad5.PGT121 led to rapid, robust, and sustained PGT121 expression in immunosuppressed Rag KO and BLT mice, whereas AAV1.PGT121 led to delayed and lower PGT121 expression. Ad5.PGT121 also led to more-durable PGT121 levels and therapeutic efficacy in BLT mice than did purified PGT121 IgG. These findings suggest that Ad vectors may have certain advantages over AAV vectors and purified IgG for *in vivo* delivery of bNAbs.

Previous studies have explored the use of purified bNAbs and AAV-vectored bNAbs for HIV-1 in animal models. To the best of our knowledge, this is the first report of an Ad vector expressing an HIV-1 bNAb. The present studies show potential advantages of Ad5.PGT121 over AAV1.PGT121 and purified PGT121 IgG in terms of rapid and durable viral load suppression in HIV-1-infected humanized mice. In contrast, AAV1.PGT121 was less effective, and purified PGT121 IgG was less durable. The rapid generation of anti-PGT121 antibody responses in immunocompetent mice, however, may limit the utility of Ad5.PGT121, although this may prove to be less of a problem in humans than in mice, since PGT121 is a human antibody. Consistent with our findings, a prior report showed more-rapid protection with Ad5 than with AAV10, encoding the anti-Bacillus anthracis protective antigen (PA) monoclonal antibody, against lethal toxin challenge (25).

There are currently multiple human antibodies with potent broadly neutralizing activity against HIV-1. These bNAbs target different epitopes on the HIV-1 envelope (Env) protein, including the CD4-binding site (26, 27), the V1/V2 and V3 glycan loops (28, 29), and the interface of gp120 and gp41 (30), and several bNAbs are being explored for the prevention and treatment of HIV-1 infection (1, 2, 31). Viral vector delivery of bNAbs provides ease of delivery and flexibility, which may be particularly useful for delivery of antibody cocktails (24, 32–35). Antiantibody responses have been reported by vectored delivery of humanized and rhesusized HIV-1-specific antibodies in rhesus monkeys (19, 20, 36) and against antibody-like immunoadhesins (37), but the extent to which this may be a clinical problem remains to be determined.

We observed viral rebound in mice that received intravenous PGT121 IgG, which correlated with the decline of serum PGT121 levels to <100 ng/ml. Rebound did not appear to be due to the emergence of resistance, consistent with previous studies with PGT121 in chronically SHIV-infected rhesus monkeys (2, 38). These data suggest that viral rebound occurred in the present study when antibody levels waned rather than as a result of virus escape (39).

For this study, we used Ad5 for proof of concept, but we have previously demonstrated that baseline Ad5 seroprevalence is extremely high in sub-Saharan Africa (40). We therefore also developed an alternate Ad serotype vector, Ad26.PGT121, with lower titers of baseline antibodies (40; data not shown). Vectored delivery of antibodies will also need to prioritize the selection of particular vectors, such as alternative serotype Ads, as well as address the challenges of antiantibody responses.

In conclusion, our data demonstrate that Ad5.PGT121 reduced viral loads in HIV-1-infected humanized mice more effectively than did AAV1.PGT121 and purified PGT121 IgG. The potential role of bNAbs in HIV-1 cure strategies is under investigation (41). Antiretroviral drugs do not eliminate the latent viral reservoir, and thus codelivery of bNAbs with latency-reversing agents represents one potential strategy (42). Our studies suggest that Ad-vectored bNAbs offer potential advantages that warrant further investigation.

## MATERIALS AND METHODS

### Viral vector construction, purification, and quantification.

Replication-deficient Ad5 vectors expressing PGT121 were constructed essentially as previously described (43). Briefly, PGT121 heavy and light chains (44) connected by an F2A linker (45) were cloned into the expression cassette of the Ad5 and Ad26 vector systems and screened by restriction enzyme analysis and sequencing. E1-complementing cells were transfected with linearized adenoviral plasmids, and homologous recombination yielded full-length, replication-deficient adenovirus that was plaque purified. A single, intact clone was expanded, and virus was purified using cesium chloride density centrifugation. The titer of the purified virus was determined by spectrophotometry, and infectivity was confirmed by PFU assay in E1-complementing cells. The presence of intact PGT121 was confirmed by sequencing over the transgene cassette.

The identical PGT121 expression cassette, consisting of a cytomegalovirus (CMV) promoter, PGT121 heavy and light chains separated by an F2A linker, and simian virus 40 (SV40) poly(A) sequence, was cloned into an AAV2-ITR plasmid. AAV1-RepCap and AAV helper plasmid pHELP (Applied Viromics, Fremont, CA) were cotransfected with AAV2-ITR-PGT121 in 293T cells plated 1 day prior to transfection. Cells were incubated at 37°C and 10% CO_2_ and harvested 72 h after transfection. Virus was released from the cells by freeze-thawing and treated with benzonase to remove cellular nucleic material. AAV1 purification was performed by iodixanol gradient centrifugation (46) followed by fast-performance liquid chromatography (FPLC) purification over a HiTrap 1-ml Mono Q Column (GE Healthcare). Fractions containing AAV1-PGT121 were confirmed by SDS-PAGE, pooled, and buffer exchanged into phosphate-buffered saline (PBS)-sucrose buffer. Packaged virus particles were quantified by RT-PCR and visualized by negative-stain electron microscopy. The presence of intact PGT121 was confirmed by sequencing over the transgene cassette.

AAV2/8 expressing luciferase (AAV2/8.Luc) was purchased from Vector Biolabs, PA.

### Mice.

Female 6- to 10-week-old C57BL/6, BALB/c, and RagKO (B6.129S7-Rag1tm1Mom/J) mice (The Jackson Laboratory, Bar Harbor, ME) were maintained in accordance with the Institutional Animal Care and Use Committee guidelines of Beth Israel Deaconess Medical Center.

### Ad, AAV, and IgG administration.

Ad and AAV vectors were administered at specified viral particle dosages in PBS by the intramuscular route in the quadriceps in a 50-μl volume. Purified PGT121 IgG was diluted in 100 μl PBS and administered by the intravenous route. After vector administration, mice were bled to determine PGT121 (or anti-PGT121) antibody concentration in the serum.

### Quantification of PGT121 and anti-PGT121 by ELISA.

A quantitative immunoglobulin ELISA for PGT121 detection was performed essentially as previously described (47). Briefly, ImmunoClear ELISA plates (Thermo Scientific) were coated with 1 μg/ml of C97ZA.012 gp140 Env trimer. Following a wash with PBS–0.05% Tween 20, wells were blocked with Blocker Casein in PBS (Pierce). Diluted serum samples were incubated with the plates for 1 h prior to further washing and the addition of a 1:1,000 dilution of goat anti-human IgG-horseradish peroxidase (IgG-HRP) (Thermo Scientific). For the PGT121 standard, gp140 Env-coated wells were spiked with PGT121 monoclonal antibody to a maximum concentration of 0.02 μg/μl, prior to incubation, washing, and addition of goat anti-human IgG-HRP. Samples and standards were developed with SureBlue TMB, and the reaction was stopped after 1.5 min with Stop solution (KPL Inc., Zettlau, Austria). Absorbance was read within 10 min of development on a spectrophotometer at an optical density at 450 to 550 nm (OD_450–550_) with SoftMax Pro GxP v5 software.

To quantify the concentration of anti-PGT121 antibodies, the quantitative murine immunoglobulin ELISA previously described (48) was used. The plates were coated with 1 μg/ml of PGT121 MAb, and mouse IgG-HRP (Southern Biotech) was used as the detection antibody.

### SPR.

Surface plasmon resonance experiments were conducted on a Biacore 3000 (GE Healthcare) instrument at 25°C utilizing HBS-EP (10 mM HEPES [pH 7.4], 150 mM NaCl, 3 mM EDTA, 0.005% P20; GE Healthcare) as the running buffer. Immobilization of protein A (Thermo Scientific) to CM5 chips was performed following the standard amine-coupling procedure as recommended by the manufacturer (GE Healthcare). RagKO mouse serum (following either Ad5.PGT121, AAV1.PGT121, or purified PGT121 IgG administration) was diluted in HBS-EP, resulting in about 600 response units (RU) of total captured IgG. Binding experiments were conducted with a flow rate of 50 μl/min with a 2-min association phase and a 5-min dissociation phase. Regeneration was conducted with one injection (3 s) of 10 mM glycine hydrochloric acid (pH 2.1) (GE Healthcare) at 100 μl/min followed by a 3-min equilibration phase in HBS-EP. Identical injections over blank surfaces were subtracted from the binding data for analysis. Binding kinetics were determined using BIAevaluation software (GE Healthcare) and the Langmuir 1:1 binding model. All samples were run in duplicate and yielded similar kinetic results. Single curves of the duplicates are shown in all figures.

### HIV-1 neutralization assay.

*In vitro* neutralization assays in luciferase reporter cells (TZM-bl cells; NIH AIDS Research and Reference Reagent Program) were performed as previously described (49), with serum samples serially diluted using 5-fold titration series. The HIV pseudoviruses tested, 6811.v7.c18, P1981_C5_3, and R2184.c04, were generated via transfection in 293T/17 cells as previously described (50).

### Adenovirus neutralization assay.

Ad5-specific neutralizing antibodies were measured using a luciferase-based neutralization assay as previously described (51). Briefly, serum from treated mice was serially diluted, Ad5.Luc virus was added, and the mixture was added to A549 cells. Following 24 h of incubation, medium was removed and 100 μl PBS and 100 μl Steady-Glo substrate (Promega) were added to wells. Luminescence was read on a Victor 3 multilabel counter (PerkinElmer), and the 90% inhibitory concentration (IC_90_) was determined (dilution of serum at which 90% of Ad5.Luc was neutralized).

### Western blotting.

Equivalent amounts of serum PGT121 IgG from Ad- and AAV-injected RagKO mice, as determined by ELISA, were brought to a total equivalent volume in naive RagKO mouse serum. Purified PGT121 IgG was added to an equivalent volume in naive RagKO mouse serum as a control. Samples were added to reducing sample buffer (Pierce), heated for 5 min at 95°C, and run on a 4 to 15% SDS-PAGE gel (Bio-Rad). Protein was transferred to a polyvinylidene difluoride (PVDF) membrane using the iBlot dry blotting system (Invitrogen), and membrane blocking was performed overnight at 4°C in PBS-T (Dulbecco's PBS + 0.2% [vol/vol] Tween 20 [Sigma] + 3% [wt/vol] bovine serum albumin). The membrane was incubated for 1 h with PBS-T containing a 1:5,000 dilution of anti-human Fc HRP (Jackson ImmunoResearch), washed, and developed using the Amersham ECL plus Western blotting detection system (GE Healthcare) and CL-Xposure film (Thermo Fisher Scientific).

### BLT humanized mice.

BLT (bone marrow/liver/thymus) humanized mice were generated as previously described (52). Briefly, NOD/SCID/IL-2Rγ^−/−^ mice were subjected to sublethal whole-body irradiation before human fetal thymus and liver fragments and CD34^+^ cells were injected. Maximal peripheral blood human T cell numbers were reached ∼14 weeks after reconstitution, with mice deemed suitable for experiments if >40% of peripheral blood lymphocytes were hCD45^+^. Mice were infected intraperitoneally (i.p.) with 10^5^ 50% tissue culture infectious doses (TCID_50_) of HIV-1 JR-CSF, to achieve 100% infection. Bleeds were obtained by submandibular puncture, and plasma was isolated and stored at −80°C. All mice were kept in accordance with Institutional Animal Care and Use Committee guidelines.

### Viral load quantification.

HIV-1 viral loads were assessed as previously described, adapted to target Gag of HIV-1 (53). Briefly, total RNA was extracted from serum with QIAcube HT (Qiagen, Germany) using the Qiacube 96 Cador pathogen HT kit and reverse transcribed using superscript III (Invitrogen). The wild-type HIV JR-CSF Gag gene (GenBank M38429.1) was utilized as a standard. RNA standards were generated using the AmpliCap-Max T 7 High Yield Message Maker kit (Cell Script, Madison, WI) and purified with the RNA Clean and Concentrator kit (Zymo Research, CA, USA). RNA quality and concentration were assessed by the BIDMC Molecular Core Facility. Log dilutions of the RNA standard were reverse transcribed and included with each RT-PCR assay. Viral loads were calculated as virus particles (vp) per milliliter. Assay sensitivity was >100 copies/ml.

### SGA.

The single genome amplification assay was adapted from Keele et al. (54). Briefly, viral RNA was isolated and reverse transcribed to viral cDNA using HIV EnvR1, 5′-TTGCTACTTGTGATTGCTCCATGT. First-round PCR was carried out with the Q5 High-Fidelity 2× master mix (NEB) together with primer HIV EnvF1, 5′-TAGAGCCCTGGAAGCATCCAGGAAG, and HIV EnvR1. PCR conditions were 1 cycle at 98°C for 30 s, 35 cycles at 98°C for 15 s, 55°C for 15 s, and 72°C for 55 s, followed by a final extension at 72°C for 2 min. A 1-μl volume of the first-round PCR product was added to the Q5 master mix with primers HIV EnvF2, 5′-TTAGGCATCTCCTATGGCAGGAAGAAG, and EnvR2, 5′-GTCTCGAGATACTGCTCCCACCC. PCR conditions were as described above but increased to 45 cycles at 98°C for 15 s. Amplicons from cDNA dilutions resulting in <30% positive were a result of a single cDNA amplification and were precluded from sequencing. For each sample, between 20 and 30 sequences were analyzed.

### *In vivo* bioluminescence.

Luciferase transgene expression through luminescence was quantified using an IVIS Series 100 imaging device and Living Image software v2.50.1 (Xenogen). At selected time points, mice were anesthetized by 1.5% isoflurane inhalation and injected i.p. with 150 μl of XenoLight RediJect d-luciferin Ultra (150 mg/kg of body weight; PerkinElmer). Imaging was performed for a 60-s exposure time, 1.2 f/stop, and medium binning.

### Statistical analyses.

Statistical analyses were performed using Prism 6.0 (GraphPad Software). Nonparametric data were analyzed by the Kruskal-Wallis test with Dunn's multiple-comparison posttest (more than two groups) or the two-tailed Mann-Whitney U test (for two groups). For parametric data, a one-way analysis of variance (ANOVA) with Bonferroni's multiple comparison posttest (more than two groups) or an unpaired *t* test (for two groups) was used. *P* values of <0.05 were considered significant (*, *P* < 0.05; **, *P* < 0.01; ***, *P* < 0.001).

### Accession number(s).

All SGA HIV-1 *env* sequences discussed in this paper have been deposited in GenBank (accession numbers MG706260 to MG706443).
